# CRAFT—A Proposed Framework for Decentralized Clinical Trials Participation in Canada

**DOI:** 10.3390/curroncol28050329

**Published:** 2021-09-30

**Authors:** Stephen Sundquist, Gerald Batist, Kathy Brodeur-Robb, Kathryn Dyck, Bernhard J. Eigl, David K. Lee, Jaqueline Limoges, Holly Longstaff, Jim Pankovich, Anna Sadura, Patrick Sullivan, Janet E. Dancey

**Affiliations:** 1 Canadian Cancer Clinical Trials Network, Toronto, ON M5G 0A3, Canada; Janet.Dancey@oicr.on.ca; 2 Segal Cancer Centre, Jewish General Hospital, McGill University, Montreal, QC H3T 1E2, Canada; gerald.batist@mcgill.ca; 3 C17 Council for Children’s Cancer and Blood Disorders, Edmonton, AB T6G 1C9, Canada; kathy.brodeur-robb@ahs.ca; 4 CancerCare Manitoba, Winnipeg, MB R3E 0V9, Canada; kdyck5@cancercare.mb.ca; 5 Division of Medical Oncology, BC Cancer, Vancouver, BC V5Z 4E6, Canada; Bernie.Eigl@bccancer.bc.ca; 6 Health Products and Food Branch, Health Canada, Ottawa, ON K1A 0K9, Canada; david.lee2@canada.ca; 7 Ontario Cancer Research Ethics Board, Toronto, ON M5G 0A3, Canada; Jacqueline.Limoges@oicr.on.ca; 8 Provincial Health Services Authority, Vancouver, BC V6H 4C1, Canada; holly.longstaff@phsa.ca; 9 Bold Therapeutics Inc., Vancouver, BC V6C 1E1, Canada; jp@bold-therapeutics.com; 10 Canadian Cancer Trials Group, Queen’s University, Kingston, ON K7L 3N6, Canada; anna.sadura@me.com; 11 Team Finn Foundation, Vancouver, BC V7K 1V4, Canada; PSullivan@wt.ca

**Keywords:** decentralized clinical trials, CRAFT, remote trial framework, trial cluster, remote trial management, remote trial access, Canada

## Abstract

Canada’s vast geography, and centralized delivery of cancer care and clinical trials create barriers for trial participation for patients in remote and rural settings. The development and implementation of a framework that enables safe and regulatory compliant trial participation through local healthcare providers would benefit Canadian patients, clinicians, trial sponsors and the health care system. To address this issue, representatives of Canada’s cancer clinical trial community met to identify key challenges and develop recommendations for remote patient participation in trials. A structured literature review identified remote/rural trial delivery models. A panel of expert stakeholders reviewed the models and participated in a workshop to assess health system readiness, identify needed processes, tools and mechanisms, and develop recommendations for a Canadian framework for decentralized clinical trial conduct. The Canadian Remote Access Framework for clinical Trials (CRAFT) represents a risk-based approach used by site investigators to delegate responsibilities for a given trial to satellite health centres within a hub-and-spoke “trial cluster”. The Framework includes specific recommendations to ensure research experience, capacity, regulatory compliance and patient safety. Canada’s cancer care and telemedicine systems can be leveraged to enable broader access to clinical trials for patients who are geographically remote from cancer centres. CRAFT’s risk-based framework is based on other successful models of remote trial patient management and is in the pilot implementation phase in Canada.

## 1. Introduction

Clinical trials are vital to improving standards of cancer care, yet participation in clinical trials is low in Canada. For cancer trials, the reported rates of trial participants to new incident cancer cases are 4.7% overall, and as low as 1% in some Canadian provinces, as compared to 14% in the UK [1,2]. This difference may partly be due to issues of access. Canada’s vast geography creates challenges that limit trial participation and the ability of patients to access innovative new therapies and treatment strategies. Over 30% of the population reside outside of large/medium population areas where regional cancer centres are located [3,4]. Additional barriers to clinical trial participation may include lack of awareness of available trial options and patient or clinician biases toward trial participation [5,6]. Given that low patient accrual is a leading reason cited for premature trial closure, the scale of unrealized accrual potential in Canada is enormous [7]. 

There are strong ethical, scientific, and medical reasons that cancer patients should have access to trials. The ethical principle of respect for persons and equity, including the just distribution of resources and of risk and benefit, requires that people, regardless of where they live, should have the opportunity to participate in clinical trials [8]. It also aligns with the necessary condition for accessibility of services identified within the Canada Health Act [9]. Involving underrepresented populations can improve generalizability of research results and broader access would improve the feasibility of novel therapeutic trials in a country with a low population density, particularly in the study of rare diseases.

Telecommunication advances and health care delivery models have enabled cancer patients to be managed closer to their homes. Telemedicine for patient assessments and extending cancer care delivery to include clinical teams at health care facilities remote from cancer centres have become standard across Canada. In addition, remote trial participation during the COVID-19 pandemic was enabled through evolving Canadian and international regulatory guidances [10,11,12]. Proof-of-concept initiatives that leverage these approaches have shown that patients can participate safely and trial conduct can be maintained with appropriate compliance and oversight [13].

## 2. Methods

In line with its objective to support the conduct of academic sponsored clinical trials, the Canadian Cancer Clinical Trials Network (3CTN) convened a steering committee of academic, public, government and industry stakeholders with expertise and knowledge of Canada’s clinical trial environment. The committee was charged to review existing models for remote access to trials; assess national health system readiness; identify needs and enabling mechanisms; and to develop recommendations that would serve as a framework for a Canadian approach (refer to Table A1 for a list of steering committee members and Supplementary Materials S1 for committee terms of reference). 

Published literature was searched using MESH terms “clinical trials” and “health services accessibility” of Medline and Embase databases to identify existing models that enabled rural and remote patients to participate in trials closer to their homes. Both publications and references were reviewed for relevance. Stakeholder interviews with trial sponsors, ethics board members, regulators, health services providers, patients and clinical trial researchers were conducted to identify relevant initiatives, case studies and existing resources to help plan, assess feasibility and support trial conduct for geographically remote patients.

Results from the publications and interviews informed the approach for a structured stakeholder workshop held at the 2019 Canadian Cancer Research Conference (see Supplementary Materials Table S1). Workshop participants included trial sponsors, experts in telemedicine, clinical trial agreements, regulatory affairs, research ethics and privacy, clinical research professionals, patient partners, as well as representatives from Health Canada. Interactive sessions were designed to obtain recommendations on framework options for remote access, requirements for implementation, and areas requiring clarification within current regulations to foster adoption (see Supplementary Materials Table S2). The draft framework and recommendations were developed by the steering committee and shared with the workshop participants and broader stakeholder community for comments before finalizing.

## 3. Results

### 3.1. Results from the Literature Review

Multiple publications recommended trial sponsors and researchers explore the use of technologies and other tools to reduce the effort, time, cost and travel burdens associated with clinical trial participation [6,14]. Models of decentralized trial conduct in Canada and Australia were identified, as were frameworks for delivering standard of care cancer treatment at local community healthcare centres via telemedicine programs [5,15].

Telemedicine and remote care are available in all provinces and can be used to conduct trial specific activities. The Federation of Medical Regulatory Authorities of Canada defines telemedicine as a medical service provided remotely via information and communication technology. “Remotely” is defined as without physical contact regardless of distances [16]. Existing technologies can support consent, document and data collection, training, oversight and compliance processes. Electronic healthcare platforms and technology advancements have enabled timelier, effective patient assessments and data collection. Compliance with privacy regulations and electronic data standards enable extension of the circle of care among health providers at different health care facilities [2,17,18]. Table 1 provides some representative examples of technology-enabled, distributed models of adult and paediatric cancer care from across Canada which can be leveraged to conduct clinical trials.

### 3.2. Two Case Studies of Remote Trial Participation

Two successful models of remote trial access are the Clinical Oncology Society of Australia’s Australasian Teletrial Model (COSA ATM) and the Pediatric Oncology Group of Ontario (POGO) Satellite Program. Both leverage telemedicine technologies and health care collaborations and provide guidelines to enable participation of remote and rural patients in clinical research [19,20]. 

#### 3.2.1. COSA Australasian Tele-Trial Model (COSA ATM)

Australia has made a significant investment in developing and implementing a model to address the low population density and vast geography to enable trial participation [21,22]. An implementation pilot project evaluated a hub and spoke model linking cancer centres to remote health care providers participating in a portfolio of industry, cooperative group and investigator-sponsored trials of different designs and interventions [23]. Table 2 provides examples of trials in the COSA ATM pilot.

A formal evaluation of trial conduct for centres participating in the randomized Phase III trial, monarchE found that the data produced was acceptable for commercially sponsored research destined for marketing applications and regulators. Furthermore, it showed successful recruitment and management of rural and remote patients to trials closer to their homes and increased clinical trials capability and training of regional sites in Good Clinical Practice (GCP) [13]. These findings are consistent with those from the pilot of the US National Cancer Institute Community Cancer Centers Program, which demonstrated both feasibility and improvement in trial recruitment at community centres on NCI sponsored trials [24]. 

#### 3.2.2. Pediatric Oncology Group of Ontario (POGO) Satellite Program

Participation in multi-centred trials is a core component of childhood cancer care. Given the small numbers of children with cancer, community hospitals cannot independently obtain and maintain the expertise, capacity or infrastructure for clinical trial activities. Since 1998, POGO’s Provincial Pediatric Oncology Satellite Program has enabled the transfer of certain aspects of a child’s clinical care, including clinical trial research activities, to community hospitals closer to the children’s homes. The POGO model is a networked, shared-care system partnership of Ontario’s five tertiary hospitals and POGO Satellite community hospitals that enables some clinical trial activities to be conducted in community settings. Industry and academic trial sponsors such as the Children’s Oncology Group (COG) and the US National Cancer Institute (NCI) endorse the model for conduct of their trials [20,25]. 

### 3.3. Recommended Model

**Figure 1:**
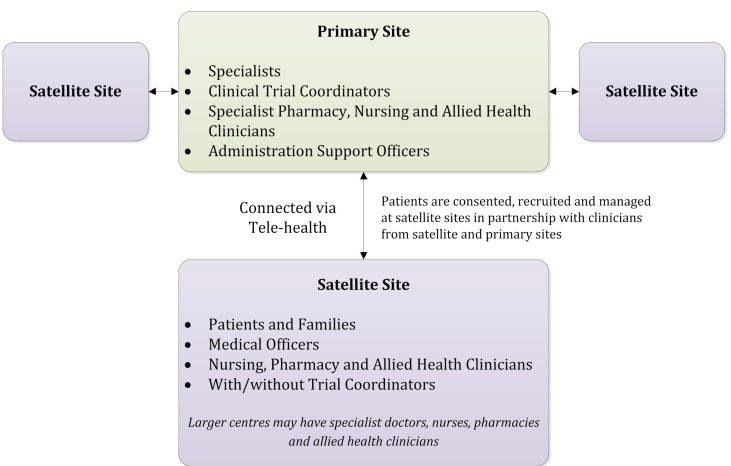
A trial cluster from the Australasian tele-trial model. Adapted from Clinical Oncology Society of Australia, Australasian Tele-Trial Model: A National Guide for Implementation. 2016.

Similarities between Canadian and Australian population distributions, national cancer centre networks, regulations and health system funding arrangements suggest the COSA ATM “hub and spoke” of lead cancer centre linked with community health care facilities and personnel is a feasible option for Canada. The model is conceptually straightforward, applicable to a wide variety of trials and scalable across regions and trial populations. When tested, it showed that patient recruitment, retention and national trial capacity was enhanced. With agreement of a study sponsor and participating institutions, a regional or metropolitan centre serving as the primary site holds overall responsibility for supervision and coordination for a hub-and-spoke “trial cluster” comprised of one or more local satellite site(s) (see Figure 1). 

Satellite sites are delegated responsibilities and collaborate on activities such as recruitment, consent, treatment and/or follow-up using a risk-based approach that considers trial complexity, patient safety, site research capacity and professional competencies. Trial procedures can be streamlined and in-person patient visits can be conducted at the cancer centre, local health centre, via telemedicine, or through compliant remote data collection platforms. Protocol-specific and core research training (e.g., GCP, TCPS 2) needs are determined based on defined research roles and provided to those personnel with identified research responsibilities. Training requirements would not necessarily formally extend to allied healthcare or administrative personnel performing duties that fall within professional education, accreditation or standards of care. 

### 3.4. Canadian Remote Access Framework for Clinical Trials (CRAFT) Recommendations

The CRAFT framework provides recommendations in eight areas, summarized in Table 3, to facilitate remote trial conduct: infrastructure, funding, trial planning and conduct, ethics, regulatory, legislative and legal requirements, indemnity and insurance, engagement and communication.

The CRAFT steering committee recommends the following be conducted to evaluate the remote access framework and guide its implementation in Canadian settings.

1. Conduct pilot studies to evaluate the model and share findings. Pilot implementation projects should build upon existing regional networks of shared clinical care with personnel that are supportive of improving trial participation. Pilot clusters would extend care delivery to include trial delivery, leveraging existing regional patterns of care and telemedicine capacity across site networks. The cluster could begin by participating in a trial of interventions of lower risk and complexity, such as a trial assessing different standard of care treatments or supportive care measures. Such a trial experience would establish the cluster, and the processes to ensure trial oversight and conduct (e.g., site contracts, REB, training, delegation of responsibilities).
2. Identify agreement terms among investigators, sub-investigators, healthcare providers (or their representatives), insurers and sponsors that can address research responsibilities, professional liability, and indemnity. Ideally, a core set of template documents could be developed to be used by those interested in implementing the model and framework.
3. Identify feasible and cost-effective options for establishing linkages between centres that consider professional capacity, existing workflows, and scheduling requirements. Research activities should be distributed to efficiently and effectively ensure optimal engagement of personnel and resources.
4. Continue to consult with Health Canada to ensure recognition and support of models of trial conduct within a cluster across regulations, guidance documents, review of clinical trial applications and site/sponsor inspections.

## 4. Discussion

The CRAFT initiative aims to develop a model for Canada that would address the geographic barriers to trial participation. Although development of the framework was initiated in 2019, prior to the COVID-19 pandemic, transformational changes in healthcare services since have helped address access restrictions imposed by the pandemic and allowed many of the key concepts of decentralized trial conduct to be implemented. For example, there is now more widespread use of distributed care models and virtual platforms for healthcare and for trial conduct, along with new regulatory measures that enable leveraging virtual care models for decentralized clinical trial (DCT) activities [26,27]. Telemedicine for virtual consults, direct-to-patient shipping of oral drugs, use of local laboratories and imaging services to limit visits to cancer centres have been implemented to ensure that patient participation on trial protocols could continue [10]. Such activities can and should continue beyond the pandemic to become routine means for trial participation.

The POGO and Australian experiences and the approaches adopted to ensure continued trial participation during the pandemic showed that remote or decentralized participation in clinical trials is feasible and preferable for many patients, health care providers and trial sponsors. The CRAFT model provides practical recommendations that if implemented, would ensure successful trial conduct. Fundamental to adoption would be recognition by sponsors, regulators, ethics boards and participating health care institutions of a “cluster” as an organizational unit for trial conduct. Trial complexity, capacity and capabilities within the cluster will determine the scope of delegated activities. Access to and sharing of trial participants’ health information as part of the “the circle of care” within the cluster is also a key consideration. The extent of training required by remote practitioners and staff should be based on whether the proposed trial activities fall within the scope of routine standard practice or are research-specific. Additional communication, oversight, SOPs and training within the cluster and provision of resources to support the activities of personnel at participating sites are needed to assure patient safety, research quality and regulatory compliance. 

Decentralized trial activities require added commitments for tools, infrastructure, communication frameworks and funds along with added time investments for site personnel. New agreements, training, study drug distribution and handling, new workflows for trial management, oversight and monitoring are implementation costs to be factored. Costs may be mitigated by improved recruitment and retention, reduced patient travel, better patient quality of life, and broader health system and economic benefits of a decentralized approach to trial delivery. Additional benefits may include more rapid trial completion, trial results that are more representative of outcomes of the intended “real world” population, and a broader health care workforce with expertise in clinical research. Higher trial accrual rates would improve trial feasibility, particularly for rare diseases. Improved overall participation rates across all trials would serve to reduce the disparity in outcomes for geographically dispersed populations.

There is a unique and timely opportunity to implement, evaluate and improve CRAFT across all regions of Canada. 3CTN plans for demonstration of CRAFT proof of concept pilot among member cancer centres. The pilot will be enabled by the CRAFT framework and the 3CTN collaborative network of clinicians, patient representatives, industry sponsors, Health Canada and other stakeholders. Scaled implementation of CRAFT across the Canadian clinical trial system will be informed by evaluation of pilot outcomes, further knowledge exchange and would also benefit from health system policy development that recognizes and enables equitable access to clinical trials as a fundamental component of standard of care delivery.

## Figures and Tables

**Table 1:** Canadian examples of oncology networks/distributed models of care.

Network	Description
Alberta’s Community Cancer Network	Comprised of two tertiary centres, four associate centres and 11 community cancer centres. Community cancer centres must satisfy eligibility criteria for safe and effective chemotherapy treatment and follow-up care. Provides treatment, psychosocial & palliative care, prevention and screening services.
The North East Regional Community Oncology Clinic Network (COCN)	Tele-oncology program operating out of Sudbury, Ontario serves a population of 600,000 spread over about 300,000 square kilometres. A regional network of fourteen regional satellite clinics offer imaging, chemotherapy and in one case, radiotherapy to 5,000 patient consults annually.
Atlantic Provinces Pediatric Hematology/Oncology Network (APPHON)	Platform for healthcare providers, patients and caregivers to access comprehensive health services and clinical care at regional centres located in Halifax, Nova Scotia and St. John’s, Newfoundland as well as education and research related to paediatric hematologic or oncologic disorders and supports for member organizations and groups in development of standards.

**Table 2:** Examples of COSA ATM portfolio trial diversity [22].

Trial	Sponsor
Abemaciclib Combined with Endocrine Therapy for the Adjuvant Treatment of HR+, HER2-, Node-Positive, High-Risk, Early Breast Cancer (monarchE)	Eli Lilly and Company
Targeted thromboprophylaxis in ambulatory patients receiving anticancer therapies (TARGET-TP)	Victorian Cancer Agency/Peter MacCallum Cancer Centre
Aspirin for Dukes C and High-Risk Dukes B Colorectal Cancers: An International, Multi-Centre, Double Blind, Randomised Placebo Controlled Phase III Trial (ASCOLT)	Australasian Gastro-Intestinal Trial Group

**Table 3:** CRAFT Recommendations.

	Framework Element (s)		Recommendations
1	Infrastructure, personnel and system development	1.1	Address human resources, equipment and facility requirements at satellite centres.
		1.2	Develop contingency plans to assure patient participation can be supported throughout the course of the clinical trial and long-term follow-up.
		1.3	Use a risk-based approach to identify protocol-specific training needs for satellite personnel that is based on the extent of delegated responsibilities and scope of practice.
		1.4	Assess what aspects of core clinical trial competency training (ICH GCP E6(2), Ethical Conduct of Research Involving Humans (TCPS 2), may be required for remote activities.
		1.5	Establish mentoring relationships with satellite personnel for professional trial competencies development.
		1.6	Provide a decision guide for risk-based assessment with criteria for establishing satellite site suitability for a trial.
		1.7	Provide templates for clinical trial budgets, agreements between the sponsor and primary site in a cluster as well as sub-agreements between the primary site and each satellite.
		1.8	Provide tools (e.g., template checklists) to inform supervision plans and roles and responsibilities for satellite activities.
2	Costs and funding requirements	2.1	Invest in applied studies and evaluations that can demonstrate feasibility for a range of trial types, patient populations, distributed care models, trial cluster configurations, etc.
		2.2	Provide financial support to primary sites to support initial costs to create infrastructure, systems, training and visits at satellite centres to set up the cluster.
3	Trial planning and conduct	3.1	Design clusters to be robust and flexible to allow the addition of satellite sites throughout the period a trial is open.
		3.2	Leverage pre-existing telemedicine/care delivery practices with satellites, when feasible.
		3.3	Engage clinicians and patients from rural and remote sites in trial design.
		3.4	Consider protocol accommodations that allow for clinical trial conduct at satellite centre.
		3.5	Adopt risk-based criteria to determine remote centres involvement in the trial. Such criteria may include complexity of trial design, product safety profile, or required protocol assessments.
		3.6	Adopt a risk-based criteria to determine activities that can be delegated to a satellite site, required staffing complement, qualifications, equipment and facilities.
4	Health Canada regulatory guidelines and inspections	4.1	Update or interpret the Health Canada Food and Drug Regulations, Part C, Division 5 “Drugs for Clinical Trials Involving Human Subjects” to recognize the required elements of the proposed framework. Specifically, that: i. A clinical trial cluster conforms to the definition of a trial site; and ii. Qualified/Principal Investigator responsibilities may be delegated to satellite clinicians and staff within the scope of each delegate’s professional practice.
		4.2	Health Canada reviews and inspections should recognize the trial cluster, delegation of Qualified Investigator responsibilities to satellite sites and assess regulatory compliance so as not to cause undue burden for the primary site or for satellite sites.
5	Ethics review	5.1	Recognize the primary site’s REB as the REB of record for the cluster so as not to introduce added steps or barriers to the ethics review process for satellite sites.
6	Patient privacy	6.1	Adopt the interpretation of the Personal Information Protection and Electronic Documents Act (PIPEDA) legislation and provincial privacy laws that recognizes healthcare professionals performing trial related activities as part of a circle of care may have access to personal health information.
7	Trial agreements, Indemnity and insurance	7.1	Trial sponsors should be willing to execute agreements with the primary site and extend terms of coverage for the scope of a primary site’s coordination of satellite centres.
8	Engagement, communications and advocacy	8.1	Develop dissemination and knowledge mobilization strategies to generating broader awareness and advocacy among sponsors, researchers, clinicians, patient communities, ethics boards and regulators that can be scaled and sustained over time.
		8.2	Create a strategy for health policy advocacy to recognize and support clinical trials as standard of care.

## Supplementary Materials

The following are available online at https://www.mdpi.com/article/10.3390/curroncol28050329/s1, Materials S1: CRAFT Steering Committee Terms of Reference, Table S1: CRAFT Workshop Agenda, Table S2: Remote Clinical Trial Conduct—Framework Considerations for discussion at CRAFT Workshop.

## Appendix A

**Table A1:** CRAFT steering and writing committee.

Member	Affiliation(s)	Title(s)
Stephen Sundquist * (Chair)	3CTN	Executive Director
Gerry Batist	Quebec-Clinical Research Organization in Cancer	Scientific Director
Kathy Brodeur-Robb *	C-17	Executive Director
Janet Dancey *	3CTN; Canadian Cancer Trials Group	Scientific Director; Executive Director
Kathryn Dyck	CancerCare Manitoba	Clinical Trials Manager
Bernie Eigl	BC Cancer	Provincial Director—Clinical Trials
David K. Lee	Health Canada—Health Products and Food Branch	Chief Regulatory Officer
Jacqueline Limoges *	Ontario Cancer Research Ethics Board	Chair
Jim Pankovich	Bold Therapeutics Inc	Executive Vice President, Clinical Development
Anna Sadura	Canadian Cancer Trials Group	Manager, Trial Management Group (retired)
Patrick Sullivan	Team Finn Foundation; 3CTN; Canadian Cancer Trials Group	Childhood Cancer Research Advocacy; Patient Representative; Research Advisor
Holly Longstaff * (Writing Committee only)	Provincial Health Services Authority	Director, Privacy and Access, PHSA Research and New Initiatives Research & Academic Services
*Writing Committee Member		
