# Supplementary material for: CRAFT—A Proposed Framework for Decentralized Clinical Trials Participation in Canada
Source: Curr Oncol. 2021 Sep 30;28(5):3857–65. doi: 10.3390/curroncol28050329 (PMC8534531; doi:10.3390/curroncol28050329)
Supplement: Supplementary file 1 [file curroncol-28-00329-s001.zip › curroncol-1360944-supplementary.pdf]

Supplementary Materials

CRAFT Steering Committee Terms of Reference

Purpose

The Steering Committee ("Steering Committee, SC") will provide expert knowledge and strategic advice in guiding the generation of a position paper outlining recommendations for improving access for cancer patients for whom distance from the nearest cancer center presents a barrier to trial participation. The purpose of the SC is to help inform the project scope, reference elements to be considered and key informants that will be required for a comprehensive review to take place in a planned stakeholder Workshop. The SC will review Workshop outcomes and assist with assessing feasibility and priority-setting for resulting recommendations, follow-up actions required and final report development.

Key Responsibilities

The Steering Committee will:

- Review and approve drafted project elements, proposed activities and identify key informants and reference sources to be considered for Workshop development
- Inform long-term strategy for stakeholder engagement through the Canadian clinical trial environment
- Identify and recommend innovative approaches to address anticipated barriers to implementation;
- Identify relevant initiatives, existing resources and/or case examples incorporating elements of remote clinical trial access which may help inform recommendations for a model framework and long-term implementation strategy that leverages existing infrastructure as much as possible, reflects stakeholder priorities and considers healthcare innovation and technology trends;
- Drafting white paper and summary recommendations for establishing a framework for improved clinical trials access that considers: relative priorities and sequencing, feasibility, enabling requirements and potential barriers

Membership & Chair

- Members will have the required knowledge and experience in aspects of clinical trial planning and conduct relevant to this work including – regulatory requirements, ethics, research unit operations, patient involvement, contracts & agreements, community healthcare;
- Committee members will represent the geographical regions of Canada as much as possible;
- There will be 6-8 members, including and at least one member from the 3CTN Executive;
- A Chair will be nominated and approved by the membership to lead planning and conduct of committee meetings and completion of project deliverables.
- Members would be expected to attend planned meetings (see ∮5.1)

- Members are expected to draw upon personal experience, representative input, references and contacts derived from the knowledge area they represent to inform discussions;
- Members are expected to be prepared for meetings, must foster an open, collaborative climate and contribute constructive input to deliberations that support project objectives.

Terms of Appointment

- Term – Members will be appointed for the planned scope of the project, from August 2019 to March, 2020. Selection of new members will be based on consultation with 3CTN funders, executive, expert advisors and by fellow Steering Committee members, as may be required.
- Authority **–** Members will function in an advisory capacity and will be called upon to approve the project plan, stakeholder workshop agenda, support the synthesis of workshop outcomes into a comprehensive set of recommendations to be summarized in the summary report/position paper. Final decision on SC recommendations or approval will be determined by majority decision, or as may be required, by the Chair.
- Withdrawal – An individual member may withdraw at any time upon written notification to the Secretariat.
- Removal – Members will serve on the Steering Committee at the discretion of the 3CTN Executive Director and may be removed or replaced, if required, by written notification.

Meetings / Quorum

- Meetings - All meetings will be scheduled to take place via teleconference/webinar, with timing based on the availability of the majority of participants and will minimally include:
- The Steering Committee will meet in September 2019 for project kick-off as well as additionally as may be required to advise on the overall project scope and support planning for the November 2019 workshop.
- A meeting will take place in the weeks immediately following the Workshop to review outcomes and guide position paper development
- As may be required to resolve any matters stemming from the Steering Committee's collective review of the position paper draft and to approve changes required for the final version.
- Quorum – A majority of members shall constitute a quorum. Steering Committee decisions will be captured and reflected for the Workshop and inform summary recommendations in the position paper.

Members will be expected to demonstrate fairness and a commitment to an in-depth evaluation of all matters under review. Discussions during meetings shall be open, frank and free-flowing. All members will have an equal status during discussions.

Compensation

Committee members will be reimbursed for reasonable travel and accommodation expenses required for meetings and workshop attendance in accordance with the 3CTN Travel and Reimbursement policies.

Secretariat

Administrative support - preparation and circulation of agendas, background reference materials and minutes - will be provided by the 3CTN Coordinating Center, in consultation with the Executive Director and Steering Committee Chair.

Table S1. CRAFT Workshop Agenda

Time	Topic
12:30 pm	Lunch - meet & greet
1:00 pm	**Welcoming Remarks**
1:00 pm	**Introductions & Workshop Objectives**<br>*Stephen Sundquist, 3CTN*
1:05 pm	**Setting the Scene: Overview of Existing Models and Lessons Learned**<br>*Session objective:*<br>• *To explore and evaluate existing models of patients participating in clinical trials remotely and how it can be applied to Canada*
1:05 pm	Cancer Clinical Trial Participation in Remote Areas: current Canadian landscape, benefits and challenges<br>*Craig Earle, Canadian Partnership Against Cancer*
1:15 pm	Existing Remote Access Models and Capabilities: overview of the Australasian Teletrials Model, Pediatric Oncology Group of Ontario and the state of Telemedicine in Canada<br>*Janet Dancey, 3CTN*<br>Open Discussion
1:50 pm	**Breakout Group Discussions**<br>*Session objectives:*<br>*To assess Canada's state of readiness by identifying enablers, capabilities, expertise and resources from the perspective of the sponsor, primary site and satellite site*<br>• *To identify solutions to meet regulatory, ethical, legal and practical requirements*<br>*To consider the patient perspective throughout the development and implementation of a framework*
1:50 pm	Breakout Discussion Set Up and Instructions<br>*Facilitator: Greg Williams*
2:00 pm	Concurrent Breakout Discussions<br>• Breakout 1: Pre-Trial Considerations, Group A<br>• Breakout 2: Pre-Trial Considerations, Group B<br>• Breakout 3: Trial Conduct Considerations, Group C<br>• Breakout 4: Trial Conduct Considerations, Group D
2:45 pm	Break
3:00 pm	**Breakout Report Out**<br>*Facilitator: Greg Williams*<br>• Report Out: Pre-Trial Considerations, Group A<br>• Report Out: Pre-Trial Considerations, Group B<br>• Report Out: Trial Conduct Considerations, Group C<br>• Report Out: Trial Conduct Considerations, Group D<br>Open Discussion
4:00 pm	Final Recommendations & Next Steps<br>*Facilitator: Greg Williams*
4:30 pm	Adjourn

Table S2. Remote Clinical Trial Conduct – Framework Considerations for discussion at CRAFT Workshop.

	**Perspectives**				
	**Sponsor**	**Sites**		**Health Canada Food & Drug Regs., Part C, Div. 5 ICH E6(R2)**	
		**Primary Site**	**Satellite Site**	**Interpretation /Guidance**	**Regulatory Change**
**General Considerations**					
Patient safety, study feasibility, risk-based oversight, data quality					
**Pre-Trial Considerations**					
Selection of Trials and Satellite Sites					
Study Feasibility Assessment					
Site Accreditation					
Satellite Site Supervision Plan					
Site Visits					
Roles and Responsibilities of Trial Staff					
Pharmacy & Pharmacy facilities					
Pathology & Radiology					
Patient Perspective, Values, Priorities					
SOPs, Study-specific Training					
Technology & Data: platforms/systems/equipment access, validation, support					
Indemnity, Insurance and CTAs					
Research Ethics Board: review & reporting					
**Trial Conduct Considerations**					
Patient Perspective, Values, Priorities					
Participant Recruitment, Consent, Screening and Enrolment					
Medication handling					
Documentation and Reporting					
Patient Reported Outcomes					

	Perspectives				
	Sponsor	Sites		Health Canada Food & Drug Regs., Part C, Div. 5<br>ICH E6(R2)	
		Primary Site	Satellite Site	Interpretation /Guidance	Regulatory Change
Managing reporting AE, SAEs					
Source Documentation and Record Retention					
Monitoring					
Equipment and Facilities					
Financial, Budget					
